# Assessment of drug content uniformity of atropine sulfate triturate by liquid chromatography–tandem mass spectrometry, X-ray powder diffraction, and Raman chemical imaging

**DOI:** 10.1186/s40780-016-0038-7

**Published:** 2016-02-10

**Authors:** Kei Moriyama, Yoichiro Takami, Natsuki Uozumi, Akiko Okuda, Mayumi Yamashita, Rie Yokomizo, Kenichi Shimada, Takashi Egawa, Takehito Kamei, Kazunobu Takayanagi

**Affiliations:** Shujitsu University, School of Pharmacy, 1-6-1 Nishigawara, Naka-ku, Okayama 703-8516 Japan; Department of Pharmacy, Kurashiki Central Hospital, 1-1-1 Miwa, Kurashiki, 710-8602 Japan

**Keywords:** Atropine sulfate, Triturate, Uniformity, Raman imaging, XRPD

## Abstract

**Background:**

Atropine sulfate is an anticholinergic agent for treatment of hypertrophic pyloric stenosis and is orally administrated as a triturate with lactose hydrate. Because of the low safety margin of atropine sulfate, triturate uniformity is a key safety factor. In this study, we assessed the uniformity of atropine sulfate in 1000-fold triturates prepared by wet mixing and dry mixing methods and discussed the cause of the difference in uniformity between two preparation methods.

**Methods:**

A 1000-fold triturate of atropine sulfate with lactose hydrate was prepared by two different methods: wet mixing and dry mixing. The wet mixing was performed according to Kurashiki Central Hospital protocol and the dry mixing was a simple physical mixing by a rocking mixer. The uniformity of atropine sulfate content in aliquots of a 1000-fold triturate with lactate hydrate was assessed by liquid chromatography–tandem mass spectrometry (LC–MS/MS) quantification. Solid-state analyses of the triturates by Raman chemical imaging and X-ray powder diffraction (XRPD) were performed to investigate the difference in uniformity.

**Results:**

The LC–MS/MS quantification showed that the uniformity of atropine sulfate in the 1000-fold triturate was excellent for wet mixing but was significantly variable for dry mixing. On the basis of the Raman chemical imaging and XRPD analyses, it was indicated that an amorphous thin film of atropine sulfate coated the surfaces of the lactose hydrate particles during wet mixing and contributed to the uniformity of the triturate. In contrast, clusters of the crystalline atropine sulfate were found in the dry mixing samples.

**Conclusion:**

The results showed that better atropine sulfate triturate uniformity was achieved using the wet mixing method rather than the dry method and the cause of the uniformity difference between two mixing methods was indicated by the multilateral assessment.

## Background

Atropine sulfate is an anticholinergic alkaloid that has been used to prevent muscarinic effects of anticholinesterases in adults [1]. It has also been used in an oral dosage form for treatment of infant hypertrophic pyloric stenosis [2]. The dose of atropine sulfate is 0.2–0.5 mg per administration for adults and decreases to approximately 0.05 mg per kg body weight for infants. Because atropine is a nonselective anticholinergic agent and toxic effects often appear even at therapeutic doses, careful administration of atropine sulfate is required.

In Kurashiki Central Hospital, a 1000-fold triturate of pure atropine sulfate with lactose hydrate is prepared for administration, with the aim of improving handling in weighing and dosing and decreasing errors in the divided weight. However, because of the large particle size of the crystalline atropine sulfate, it is believed that simple dry mixing with lactose hydrate causes poor uniformity of atropine sulfate in the triturate, and consequently, wide variation in the amount of drug that is actually dosed. To overcome this problem, some institutions including Kurashiki Central Hospital prepare the triturate using a wet mixing method: the crystalline atropine sulfate is dissolved in water followed by mixing of the solution with lactose hydrate and drying.

The uniformity of an active pharmaceutical ingredient (API) in the triturate is undoubtedly a key factor in the safety of low-dose formulations with low safety margins, such as that for atropine sulfate. Although the uniformity of the triturate generally has been assessed by liquid-phase quantification of the API in an aliquot of triturate, solid-phase chemical imaging techniques such as Raman chemical imaging can provide more detail and direct information on the uniformity of the triturate because the particle size and dispersion state of the API can easily be shown by the imaging [3–8].

In this study, we compared the uniformity of the API in a 1000-fold triturate of atropine sulfate with lactose hydrate prepared by two different methods: wet mixing and dry mixing, using liquid chromatography–tandem mass spectrometry (LC–MS/MS) quantification of atropine sulfate [9]. To investigate the differences in the solid-state properties, such as the crystallinity and dispersion, we analyzed the triturates using Raman chemical imaging and X-ray powder diffraction (XRPD).

## Methods

### Preparation of triturate of atropine sulfate I: Wet mixing method

10-fold triturate was prepared as follows. A 1.0 g amount of atropine sulfate (Pfizer Japan Inc., Tokyo, Japan) was dissolved in 1 mL of water. To the solution, 9.0 g of lactose hydrate was added and mixed. The mixture was then dried at 60 °C for 5 h and sieved on 80 mesh. 100-fold triturate was prepared similarly and 1000-fold triturate was obtained by diluting 100-fold triturate with lactose hydrate.

### Preparation of triturate of atropine sulfate II: Dry mixing method

10-fold triturate was prepared as follows. To 1.0 g of atropine sulfate, 9.0 g of lactose hydrate was added, and the whole powder was mixed using RM-10 rocking mixer (Aichi Electric Co., Ltd., Aichi, Japan) for 30 min. The rotation and rocking speeds were 75 rpm and 10 rpm, respectively. 100-fold triturate was prepared similarly and 1000-fold triturate was obtained by diluting 100-fold triturate with lactose hydrate.

### LC–MS/MS quantification

A 250 mg aliquot of the 1000-fold atropine sulfate triturate was dissolved and diluted to obtain a 50.0 ng/mL atropine sulfate aqueous solution. The HPLC system of LC–MS/MS was an Agilent 1100 series (Agilent, Santa Clara, CA, USA) with an analytical column (CAPCELL PAK C18 MGIII S-5, 100 mm × 2.0 mm i.d., SHISEIDO, Tokyo, Japan). The isocratic mobile phase consisted of a 40:60 (v/v) mixture of 0.1 % (v/v) formic acid and acetonitrile. The flow rate of the mobile phase was 0.3 mL/min. Mass spectrometric detection was performed on a PE Sciex API 2000 spectrometer equipped with an APCI source (AB Sciex, Toronto, Canada).

### Raman and XRPD analyses

Raman chemical imaging for triturates of atropine sulfate was performed on an inVia Raman microscope system (Renishaw Plc., Gloucestershire, UK) under a 785 nm excitation laser. Powder sample was spread evenly on a glass plate, and Raman chemical image was recorded (10 mm × 10 mm area, 42.5 μm spatial resolution). Discrimination of atropine sulfate and lactose hydrate was performed according to direct classical least squares modelling [10]. The XRPD patterns were acquired on a Rigaku MultiFlex diffractometer (Rigaku Corporation, Tokyo, Japan). The 2*θ* range was 5°–70° at a 0.02° pitch.

## Results and Discussion

**Fig. 1:**
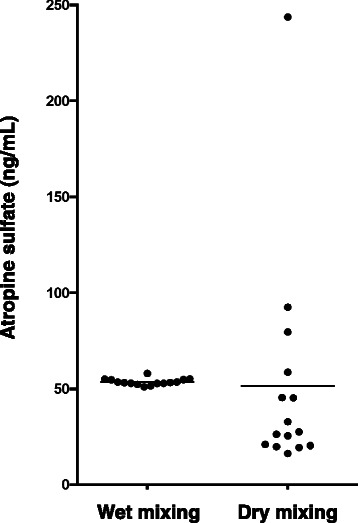
Variations in atropine sulfate concentrations in aliquots of the 1000-fold triturate prepared by two methods. Solid circles and bars indicate the individual concentrations of atropine sulfate detected by liquid chromatography–tandem mass spectrometry and the mean value, respectively, for each preparation method

The content of atropine sulfate in a 250 mg portion of the 1000-fold triturate was measured by LC–MS/MS. Figure 1 shows the variation in the detected concentrations of atropine sulfate in the triturates from each preparation method. The 15 samples from the wet mixing method showed very small variations in the 50.0 ng/mL concentrations of atropine sulfate (mean ± SD, 53.7 ± 1.7 ng/mL). The 15 samples from the dry mixing method showed a high degree of variability that ranged from 16.0 ng/mL to 243 ng/mL (51.6 ± 55.9 ng/mL), although the mean was close to the calculated value. The sample variance of the dry mixing method was significantly greater than that of the wet mixing method (*F*-test, α < 0.01). This result indicated that the 1000-fold triturate of atropine sulfate prepared by the wet mixing method was very uniform; whereas, dry mixing caused wide variation in the API content of the triturate. Considering the safety of the low-dose atropine sulfate triturate preparation with a low safety margin, the results indicated that the wet mixing method would be preferred over a simple physical dry mixing method to minimize the chance of overdosing, which incurs systemic adverse reactions such as tachycardia, blood pressure elevation, restlessness, irritability, and delirium.

**Fig. 2:**
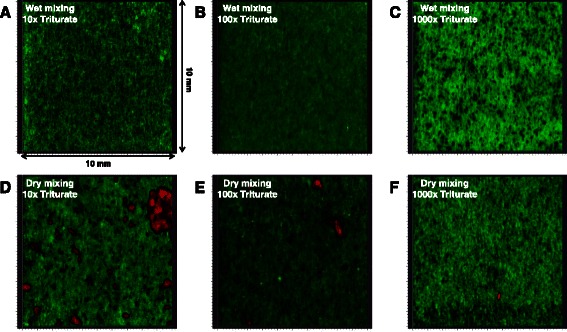
Raman chemical imaging of 10-, 100-, and 1000-fold triturates prepared by the wet or dry mixing method (10 mm × 10 mm, 42.5 μm spatial resolution). Panels **a**, **b**, and **c** represent the image of 10-, 100-, and 1000-fold triturates by the wet mixing, respectively. Panels **d**, **e**, and **f** represent the image of 10-, 100-, and 1000-fold triturates by the dry mixing, respectively. Red and green areas indicate atropine sulfate and lactose hydrate, respectively

**Fig. 3:**
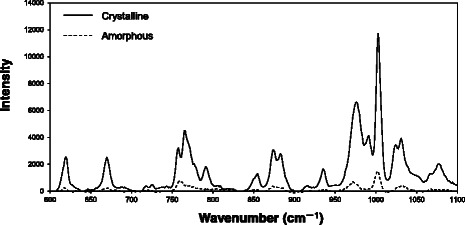
Raman spectra of the crystalline and amorphous forms of atropine sulfate

Next, to understand the dispersion of chemicals in solid samples, we applied Raman chemical imaging to triturates prepared by two methods. Interestingly, atropine sulfate was rarely detected by Raman chemical imaging in a 10 mm × 10 mm area of triturates prepared by the wet mixing method (Fig. 2a–c). On the other hand, the triturates prepared by the dry mixing method showed obvious clusters of atropine sulfate (Fig. 2d–f). The large variance in API content in the 250 mg aliquot of the 1000-fold triturate can be easily explained by the existence of clusters of atropine sulfate. However, the question of why the Raman signal of atropine sulfate disappeared in the wet mixing samples needs to be explained. We hypothesized that the crystalline atropine sulfate was transformed to the amorphous state during the wet mixing procedure and thoroughly coated the surface of the lactose hydrate particles as a thin film. Amorphous atropine sulfate exhibited a much lower Raman signal intensity than the crystalline form (Fig. 3); therefore, it was supposed that the peaks in the spectrum of the thin film of the amorphous atropine sulfate on lactose hydrate would be hidden by the much stronger intensities of the Raman peaks of the underlying lactose hydrate.

**Fig. 4:**
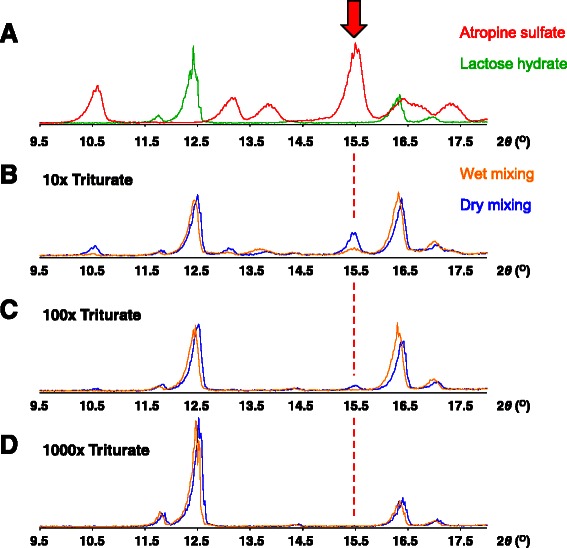
X-ray powder diffraction (XRPD) patterns of the standard sample and triturates. **a** XRPD patterns of the crystalline atropine sulfate and lactose hydrate. Red arrow indicates the distinctive diffraction signal of the crystalline atropine sulfate. **b**–**d** XRPD patterns of the 10-, 100-, and 1000-fold triturates prepared by the wet or dry mixing methods

To prove this, we performed XRPD to assess the crystallinity of atropine sulfate in the triturates. Crystalline atropine sulfate exhibited a distinctive diffraction signal at 15.5° (Fig. 4a). In the 10-fold triturate, the signal of the crystalline atropine sulfate was much stronger in the dry mixing than in the wet mixing (Fig. 4b). This means that the amorphous atropine sulfate, which showed no striking diffraction signal in XRPD, was the majority in the wet mixing samples. The same tendency was found in the 100-fold triturate (Fig. 4c), although no signal of atropine sulfate was detected in the 1000-fold triturates because the content of atropine sulfate was too low (Fig. 4d). The XRPD results strongly support the hypothesis that amorphous atropine sulfate resulted from the wet mixing method.

## Conclusions

In this study, we found that much higher uniformity of atropine sulfate in the triturate was achieved using the wet mixing preparation method of Kurashiki Central Hospital than using the dry mixing method. Solid-state analyses by Raman chemical imaging and XRPD indicated that in the triturate prepared by wet mixing, the atropine sulfate transformed into the amorphous form and coated the surfaces of the particles of the excipient. On the other hand, the dry mixing preparation method caused biased dispersion of atropine sulfate, with large clusters of crystalline drug particles. The solid-state analytical findings consistently explained why wet mixing preparation of the triturate resulted in higher uniformity of the API. These analyses can provide evidence or hints regarding the cause of uniformity and can contribute to development of better triturate preparation methods in pharmacy for low-dose and low safety margin drugs and to safer medications.
